# Changes in prevalence of obesity and high waist circumference over four years across European regions: the European male ageing study (EMAS)

**DOI:** 10.1007/s12020-016-1135-y

**Published:** 2016-10-13

**Authors:** Thang S. Han, Elon Correa, Michael E. J. Lean, David M. Lee, Terrence W. O’Neill, György Bartfai, Gianni Forti, Aleksander Giwercman, Krzysztof Kula, Neil Pendleton, Margus Punab, Martin K. Rutter, Dirk Vanderschueren, Ilpo T. Huhtaniemi, Frederick C. W. Wu, Felipe F. Casanueva, and the EMAS Study Group

**Affiliations:** 1 Institute of Cardiovascular Research, Royal Holloway, Egham & Ashford and St Peter’s NHS Foundation Trust, University of London (ICR2UL), Surrey, UK; 2 Andrology Research Unit, The University of Manchester, Manchester, UK; 3 School of Computing, Science and Engineering, University of Salford, Salford, UK; 4 Department of Human Nutrition, University of Glasgow, Glasgow, UK; 5 Cathie Marsh Institute for Social Research, School of Social Sciences, The University of Manchester, Manchester, UK; 6 Arthritis Research UK Centre for Epidemiology & NIHR Manchester Musculoskeletal Biomedical Research Unit, Manchester Academic Health Science Centre, The University of Manchester, Manchester, UK; 7 Department of Obstetrics, Gynaecology and Andrology, Albert Szent-György Medical University, Szeged, Hungary; 8 Endocrinology Unit, University of Florence, Florence, Italy; 9 Reproductive Medicine Centre, Skåne University Hospital, University of Lund, Lund, Sweden; 10 Department of Andrology and Reproductive Endocrinology, Medical University of Łódź, Łódź, Poland; 11 Institute of Brain Behaviour and Mental Health, Salford Royal NHS Trust, University of Manchester, Salford, UK; 12 Andrology Unit, United Laboratories of Tartu University Clinics, Tartu, Estonia; 13 The Endocrinology and Diabetes Research Group, Institute of Human Development, Department of Medical and Human Sciences, University of Manchester, Manchester, UK; 14 Manchester Diabetes Centre, Manchester Academic Health Science Centre, Central Manchester University Hospitals NHS Foundation Trust, Manchester, UK; 15 Department of Andrology and Endocrinology, Catholic University of Leuven, Leuven, Belgium; 16 Department of Surgery and Cancer, Imperial College London, Hammersmith Campus, London, UK; 17 Department of Medicine, CIBER de Fisiopatologia Obesidad y Nutricion (CB06/03), Instituto Salud Carlos III, Complejo Hospitalario Universitario de Santiago (CHUS), Santiago de Compostela University, Santiago de Compostela, Spain

**Keywords:** Obesity, Fat distribution, Environment, Body mass index, Waist circumference, Health promotion

## Abstract

Diversity in lifestyles and socioeconomic status among European populations, and recent socio-political and economic changes in transitional countries, may affect changes in adiposity. We aimed to determine whether change in the prevalence of obesity varies between the socio-politically transitional North-East European (Łódź, Poland; Szeged, Hungary; Tartu, Estonia), and the non-transitional Mediterranean (Santiago de Compostela, Spain; Florence, Italy) and North-West European (Leuven, Belgium; Malmö, Sweden; Manchester, UK) cities. This prospective observational cohort survey was performed between 2003 and 2005 at baseline and followed up between 2008 and 2010 of 3369 community-dwelling men aged 40–79 years. Main outcome measures in the present paper included waist circumference, body mass index and mid-upper arm muscle area. Baseline prevalence of waist circumference ≥ 102 cm and body mass index ≥ 30 kg/m^2^, respectively, were 39.0, 29.5 % in North-East European cities, 32.4, 21.9 % in Mediterranean cities, and 30.0, 20.1 % in North-West European cities. After median 4.3 years, men living in cities from transitional countries had mean gains in waist circumference (1.1 cm) and body mass index (0.2 kg/m^2^), which were greater than men in cities from non-transitional countries (*P* = 0.005). North-East European cities had greater gains in waist circumference (1.5 cm) than in Mediterranean cities (*P* < 0.001). Over 4.3 years, the prevalence of waist circumference ≥ 102 cm had increased by 13.1 % in North-East European cities, 5.8 % in the Mediterranean cities, 10.0 % in North-West European cities. Odds ratios (95 % confidence intervals), adjusted for lifestyle factors, for developing waist circumference ≥ 102 cm, compared with men from Mediterranean cities, were 2.3 (1.5–3.5) in North-East European cities and 1.6 (1.1–2.4) in North-West European cities, and 1.6 (1.2–2.1) in men living in cities from transitional, compared with cities from non-transitional countries. These regional differences in increased prevalence of waist circumference ≥ 102 cm were more pronounced in men aged 60–79 years than in those aged 40–59 years. Overall there was an increase in the prevalence of obesity (body mass index  ≥ 30 kg/m^2^) over 4.3 years (between 5.3 and 6.1 %) with no significant regional differences at any age. Mid-upper arm muscle area declined during follow-up with the greatest decline among men from North-East European cities. In conclusion, increasing waist circumference is dissociated from change in body mass index and most rapid among men living in cities from transitional North-East European countries, presumably driven by economic and socio–political changes. Information on women would also be of value and it would be of interest to relate the changes in adiposity to dietary and other behavioural habits.

## Introduction

Obesity is the major driver for type 2 diabetes mellitus, premature cardiovascular disease, the metabolic syndrome and a host of other secondary health complications [1, 2]. Furthermore recent studies indicate that obesity is a risk factor of cancer incidence at several cancer sites [3, 4]. Recognising the scale of consequent disability and healthcare costs, obesity prevention has been declared as a major public health priority in many countries [5]. The global obesity epidemic, over the last 30–40 years, can only have emerged through changes in environmental and social factors which have resulted in poor diet (high energy/low nutrient-density foods and meals), and physical inactivity [6]. Previously largely restricted to the wealthy, social deprivation has emerged as a powerful driver of obesity in the post-industrial period. Familial patterning is well recognised, but classical genetics plays only a small part [7]. It is increasingly recognised that shared environments within families serve a dual role, not only producing conditions which favour immediate positive energy balance, but also changing susceptibility to weight gain and obesity throughout the lifespan through epigenetic modifications, altering gene expressions after exposure to adverse environment in utero or in early life [8].

Recent cross-sectional analyses of European Male Ageing Study (EMAS) baseline data have shown that obesity was significantly associated with social and lifestyle factors including smoking status, physical activity level and education attainment in European men aged 40–79 years [9]. This EMAS cohort comprises populations from eight major cities, covering the Mediterranean (Spain and Italy), North-West (NW) (Belgium, Sweden and England) and North-East (NE) (Poland, Hungary and Estonia) European regions, which are diverse in lifestyle and dietary habits [10–12]. These eight cities also embody the contrasting socio-political systems between transitional and non-transitional regions in Europe [13]. Lifestyle and dietary habits in many transitional countries in NE Europe have recently been increasingly more westernized, particularly among younger people, practising an increase in consumption of calorie-dense/poor-nutrient diet, replacing healthy native diet of fruit, vegetables, nuts and grains [14]. A survey of adults aged 18–74 in the European Union conducted between 2006 and 2010 showed the prevalence of obesity (body mass index, BMI ≥ 30 kg/m^2^) in the EMAS regions was 11.3 % in Italy, 17.0 % in Spain, 13.3 % in Belgium, 22.1 % in the UK, 17.3 % in Poland, 21.4 % in Hungary and 16.0 % in Estonia [15]. More recent WHO data on adult (≥18 years) male obesity prevalence in 2014 show 22.8 % in Spain, 20.4 % in Italy, 22.3 % in Belgium, 22.5 % in Sweden, 26.9 % in UK, 24.6 % in Poland, 24.4 % in Hungary and 22.2 % in Estonia [16]. A large cross-country survey of 22,777 Europeans aged over 50 years in 2004 suggested that regional differences in prevalence of obesity were not explained by socio-demographic differences. The authors suggested diet culture, physical activity and other lifestyle behaviours may be the contributing factors [17]. These data are though based on cross-sectional surveys, with variable response rates and representativeness. Data in EMAS was collected in a standardised way across the eight countries, with relatively high response rates and permitted longitudinal comparison of changes in the prevalence of obesity and body fat distribution, indicated by BMI and waist circumference (WC), in diverse regions of the European Union. We hypothesised that changes in the prevalence of obesity may be greater in cities from the transitional countries from NE Europe than cities from countries in the Mediterranean and NW Europe.

## Methods

### Participants and study design

The study design and recruitment strategy for EMAS have been described previously [18, 19]. Briefly, an age-stratified sample of 3369 men aged 40–79 (mean ± SD: 60 ± 11) years was recruited randomly from population registers in eight European centres, five from non-transitional (Florence, Italy; Leuven, Belgium; Malmö, Sweden; Manchester, UK; Santiago de Compostela, Spain) and three from transitional countries (Łódź, Poland; Szeged, Hungary; Tartu, Estonia). Participants were assessed at baseline (2003–05) and a median of 4.3 (range 3.0–5.7) years later at follow-up survey (2008–10) [18, 19]. Ethical approval for the study was obtained in accordance with local requirements in each centre. All participants provided written informed consent.

### Lifestyle, socio–economic history and anthropometry

Participants answered questions about lifestyle habits (smoking, alcohol consumption, physical activity) and socioeconomic status (education level and employment status). Body weight and height were measured by electronic scales (SECA, Hamburg, Germany) and stadiometer (Holtain, Crymych, UK) and BMI was calculated as weight/height^2^ (kg/m^2^). WC was measured at the level between the lowest ribs and anterior suprailiac crest and mid-upper arm circumference (MUAC) at midpoint between the acromion and olecranon using non-stretchable tape measure, and triceps skinfold thickness in mm at the MUAC level using skinfold calipers (Holtain, Crymych, UK) [20]. Physical function was assessed using Physical Activity Scale for the Elderly (PASE) questionnaire score. Mid-upper arm muscle area was estimated from the equation: MUAC – (3.14 × triceps skinfold thickness) [21].

### Statistical analysis

Statistical analyses were conducted using STATA SE version 13.1 (Stata Corp, College Station, TX, USA). Individuals were categorised according to their WC ‘Action Levels’ (<94, 94–101.9 and ≥102 cm) [22] or BMI (<25, 25–29.9 and ≥30 kg/m^2^) [23]. For the purpose of this analysis, peak education attainment level was classified as below high school, or college/university; employment status as either currently in or not in employment; smoking status as never, former or current; and alcohol consumption as non-drinkers, infrequent alcohol drinkers (1–4 days/week) or frequent alcohol drinkers (≥5 days/week). Because we did not have reliable information on retirement status, we decided to classify subjects into employment and non-employment groups for men below 65 years old (working age in most countries). Physical activity levels were classified according to quartiles of PASE questionnaire score [9].

#### Development of large WC and high BMI

Changes between all BMI and WC categories, in both directions over a median 4.3 years were analysed. The development of, or the new occurrence of obesity (WC ≥ 102 cm or BMI ≥ 30 kg/m^2^) was defined as new cases who moved upwards from any lower WC or BMI categories. Those who were obese at baseline were not included in the analysis. For new development of the moderately elevated middle categories, only subjects who moved upwards from the lowest category were included, i.e., those who lost weight from the highest WC category or highest BMI category into middle category were not considered as new cases.

Analysis of variance (ANOVA) was used to assess the statistical significance of changes in WC or BMI (continuous variables) between study groups. Bonferroni multiple-comparison test was used for simultaneous estimation of paired group differences. Logistic regression analysis was used to determine whether the risk of developing obesity varied by cities, with Santiago de Compostela, Spain as the referent group; by region, with Mediterranean cities as the referent group and by transitional region status with the cities from non-transitional countries as referent. Analyses were further repeated separately in two age categories to assess whether the incidence of obesity were different for men aged 40–59 years and 60–79 years. Data were presented both unadjusted and adjusted for baseline socioeconomic status and lifestyle factors mentioned above.

## Results

**Fig. 1:**
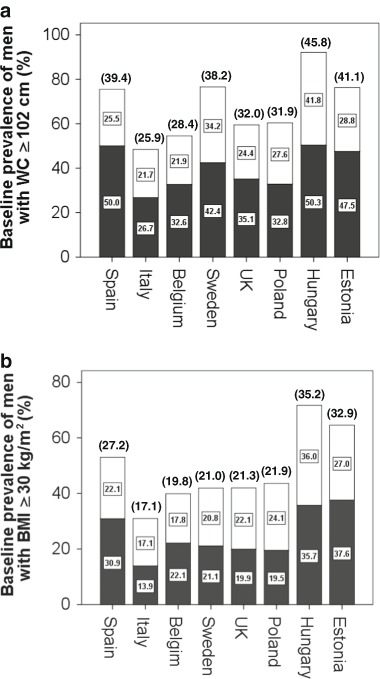
Baseline prevalence men with large waist circumference (**a)** or with high BMI (**b)** in eight European cities

Figure 1a shows that at baseline, the prevalences of WC ≥ 102 cm and BMI≥30 kg/m^2^, respectively, were highest among men living in Szeged, Hungary (45.8 and 35.2 %) and Tartu, Estonia (41.1 and 32.9 %) and lowest among men living in Florence, Italy (25.9 and 17.1 %) and Leuven, Belgium (28.4 and 19.8 %). Within each of these cities, these prevalences were higher among men over 60 than those under 60 years-old. Regionally, the baseline prevalences of WC ≥ 102 cm and BMI ≥ 30 kg/m^2^, respectively, were 32.4 and 21.9 % in Mediterranean cities (Santiago de Compostela and Florence) and similarly 30.0 and 20.1 % in NW European cities (Leuven, Malmö and Manchester), while the respective values were almost 10 % higher (*P* < 0.001) in cities from the transitional NE European (Łódź, Szeged, and Tartu) region (39.0 and 29.5 %).

Changes between all BMI and WC categories over a median 4.3 years are shown in Table 1. Increase in prevalence or development of WC ≥ 102 cm was 12.3 % (266/2148) or BMI ≥ 30 kg/m^2^ was 5.9 % (149/2499). New development of the moderately elevated middle categories of WC 94–101 cm was 16.9 % (194/1142) or BMI 25–29 kg/m^2^ was 15.1 % (132/870). Those who lost weight from the highest WC (≥102 cm: 124/1171, 10.5 %) category or highest BMI (≥30 kg/m^2^: 77/814, 9.4 %) category into middle category were not considered as new cases. There were significantly greater proportions of men living in NE European cities (transitional regions) gaining WC from lower categories (<94 cm and 94–101 cm) to highest category (≥102 cm) than men living in Mediterranean cities and in NW European cities (non-transitional countries). Conversely significantly greater proportions of men living in cities from non-transitional countries had WC reduced from moderate category (94–101 cm) to lowest category. In contrast to observations on changes in WC, there were no regional differences in the gains/reductions of BMI (data not shown).

**Table 1:** Movements of men between categories of BMI or WC from baseline to different categories at follow-up

	Proportion of men in WC < 94 cm category at baseline moved up to WC 94–101 cm category at follow-up	Proportion of men in WC < 94 cm category at baseline moved up to WC ≥ 102 cm category at follow-up	Proportion of men in WC 94–101 cm category at baseline moved down to WC < 94 cm category at follow-up	Proportion of men in WC 94–101 cm category at baseline moved up to WC ≥ 102 cm category at follow-up	Proportion of men in WC ≥ 102 cm category at baseline moved down to WC < 94 cm category at follow-up	Proportion of men in WC ≥ 102 cm category at baseline moved down to WC 94–101 cm category at follow-up
Mediterranean cities (REF)	23.2	1.1	13.2	11.0	0.0	11.4
NW European cities	20.2	2.9	12.0	20.8	1.0	8.4
NE European cities	23.3	5.7	7.4	23.5	1.0	8.0
Group differences	*P* = 0.543	*P* = **0.034**	*P* = **0.024**	*P* < **0.001**	*P* = 0.257	*P* = 0.217
Cities from non-transition region (REF)	21.3	2.3	12.6	16.9	0.6	9.7
Cities from transition region	23.3	5.7	7.4	23.5	1.0	8.0
Group differences	*P* = 0.559	*P* = **0.029**	*P* = **0.010**	*P* = **0.005**	*P* = 0.650	*P* = 0.343

	Proportion of men in BMI < 25 kg/m^2^ category at baseline moved up to “BMI 25–29 kg/m^2^ category” at follow-up	Proportion of men in BMI < 25 kg/m^2^ category at baseline moved up to BMI ≥ 30 kg/m^2^ category at follow-up	Proportion of men in BMI 25–29 kg/m^2^ category at baseline moved down to BMI < 25 kg/m^2^ category at follow-up	Proportion of men in BMI 25–29 kg/m^2^ category at baseline moved up to BMI ≥ 30 kg/m^2^ category at follow-up	Proportion of men in BMI ≥ 30 kg/m^2^ category at baseline moved down to BMI < 25 kg/m^2^ category at follow-up	Proportion of men in BMI ≥ 30 kg/m^2^ category at baseline moved down to BMI 25–29 kg/m^2^ category at follow-up
Mediterranean cities (REF)	25.3	0.8	5.0	7.6	0.0	8.9
NW European cities	17.6	0.0	9.0	8.2	0.8	8.7
NE European cities	19.1	1.2	6.1	8.8	0.3	7.8
Group differences	*P* = 0.136	*P* = 0.263	*P* = **0.020**	*P* = 0.780	*P* = 0.368	*P* = 0.875
Cities from non-transition region (REF)	20.3	0.3	7.4	7.9	0.5	8.8
Cities from transition region	19.1	1.2	6.1	8.8	0.3	7.8
Group differences	*P* = 0.816	*P* = 0.503	*P* = 0.375	*P* = 0.607	*P* = 1.000	*P* = 0.700

Group differences were determined by ANOVA or Chi-squared test

*WC* waist circumference, *REF* referent

Compared to men living in Santiago de Compostela, who had the lowest rise in WC, men living in all three cities from transitional countries showed significantly greater rises in mean WC (1.6–2.0 cm), as did men in Leuven, but not in Malmö or Manchester from the non-transitional region (Table 2A). Men living in cities from the transitional countries had greater gain in WC (1.1 cm) (Table 2A) and in BMI (0.2 kg/m^2^) (Table 2B) than those in cities from the non-transitional countries, and a significantly greater gain in WC (1.5 cm) (but not BMI) than men living in the Mediterranean cities. For both age groups below and above 60 years, compared with men living in Mediterranean cities, those living in NE European cities lost significantly more lean body tissue, estimated from MUAC (Table 2C).

**Table 2A:** A 4.3-year change in WC in eight European **cities**, and differences in gain in WC between men living in cities from the Mediterranean, NW and NE Europe, and between men living in **cities** from non-transitional (Mediterranean+NW Europe) and cities from transitional countries (NE Europe)

	*n*	Baseline mean (SD) of WC	Absolute (%) change	ANOVA for differences between cities and groups of cities in different regions		
				Between individual cities	Between Regions	Between transitional status (non-transitional/transitional)
Mediterranean: Santiago de Compostela, Spain	267	99.0 (9.5)	+0.22 (+0.25)	Referent	Referent	Referent
Mediterranean: Florence, Italy	351	96.2 (9.5)	+0.85 (+0.97)	+0.63		
NW Europe: Leuven, Belgium	381	96.2 (11.2)	+2.02 (+2.18)	+1.80*	+0.62	
NW Europe: Malmö, Sweden	356	99.9 (10.5)	+0.65 (+0.69)	+0.43		
NW Europe: Manchester, UK	312	97.6 (9.8)	+0.84 (+0.90)	+0.62		
NE Europe: Łódź Poland	310	98.5 (10.2)	+2.22 (+2.36)	+1.99*	+1.50*	+1.10*
NE Europe: Szeged, Hungary	347	101 (11.7)	+1.79 (+1.92)	+1.57*		
NE Europe: Tartu, Estonia	296	99.3 (13.4)	+2.27 (+2.36)	+2.05*		
				^a^ *P* < 0.001	^*a*^ *P < 0.001*	^a^ *P* = 0.005

Bold values indicate “statistical significant” results

*ANOVA* analysis of variance, *SD* standard deviation, *WC* waist circumference

*P < 0.05

^a^ Bonferroni multiple-comparison test. Differences from referent group

**Table 2B:** A 4.3-year change in BMI in eight European cities, and differences in gain in BMI between men living in cities from the Mediterranean, NW and NE Europe, and between men living in non-transitional (Mediterranean + NW Europe) and cities from transitional countries (NE Europe)

	*n*	Baseline mean (SD) of BMI	Absolute (%) change	ANOVA for differences between cities and groups of cities in different regions		
				Between individual cities	Between Regions	Between transitional status (non-transitional/transitional)
Mediterranean: Santiago de Compostela, Spain	272	28.2 (3.7)	−0.02 (−0.08)	Referent	Referent	Referent
Mediterranean: Florence, Italy	346	27.0 (3.6)	+0.23 (+1.05)	+0.25		
NW Europe: Leuven, Belgium	372	27.1 (3.9)	+0.10 (+0.46)	+0.12	−0.08	
NW Europe: Malmö, Sweden	344	27.2 (3.9)	+0.16 (+0.65)	+0.18		
NW Europe: Manchester, UK	300	27.6 (3.6)	−0.15 (−0.54)	−0.13		
NE Europe: Łódź Poland	310	27.5 (3.8)	+0.56 (+2.10)	+0.57*	+0.15	+0.20*
NE Europe: Szeged, Hungary	347	28.7 (4.3)	+0.16 (+0.68)	+0.18		
NE Europe: Tartu, Estonia	299	28.5 (5.0)	+0.11 (+0.42)	+0.13		
				^a^ *P* < 0.001	^a^ *P* = 0.01	^a^ *P* = 0.005

Bold values indicate “statistical significant” results

*ANOVA* analysis of variance, *BMI* body mass index, *SD* standard deviation

*P < 0.05

^a^ Bonferroni multiple-comparison test. Differences from referent group

**Table 2C:** A 4.3-year change in mid-upper arm muscle area (MUAC – 3.14 × triceps skinfold thickness) in eight European cities, and differences in mid-upper arm muscle area changes between men living in cities from the Mediterranean, NW and NE Europe, and between cities from non-transitional (Mediterranean + NW Europe) and transitional (NE Europe) in middle-aged and elderly men

	ANOVA for differences between countries and groups of countries in different regions							
	Middle-aged men (40–59 years)				Elderly men (60–79 years)			
	Absolute (%) change (SD)	Between individual cities	Between Regions	Between Transitional status (non-transitional/transitional)	Absolute (%) change (SD)	Between individual cities	Between Regions	Between Transitional status (non-transitional/transitional)
		*F*-value (*P*)	*F*-value (*P*)	*F*-value (*P*)		*F*-value (*P*)	*F*-value (*P*)	*F*-value (*P*)
Mediterranean: Santiago de Compostela, Spain	−2.6 (8.3)	Referent	Referent	Referent	−3.9 (7.6)	Referent	Referent	Referent
Mediterranean: Florence, Italy	0.0 (6.6)	**9.2 (0.002)** ^a^			−1.1 (6.0)	3.5 (0.061)		
NW Europe: Leuven, Belgium	1.0 (4.4)	**53.9 (**<**0.001)** ^a^	**43.7 (**<**0.001)** ^a^		−0.9 (5.1)	**45.4 (**<**0.001)** ^a^	**58.2 (**<**0.001)** ^a^	
NW Europe: Malmö, Sweden	0.0 (7.1)	**48.6 (**<**0.001)** ^a^			−0.6 (8.1)	**36.5 (**<**0.001)** ^a^		
NW Europe: Manchester, UK	−1.1 (4.4)	**55.7 (**<**0.001)** ^a^			−1.5 (5.4)	**38.7 (**<**0.001)** ^a^		
NE Europe: Łódź Poland	−5.1 (3.9)	**126.4 (**<**0.001)** ^a^	**15.5 (**<**0.001)** ^a^	0.01 (0.915)	−5.3 (5.7)	**57.8 (**<**0.001)** ^a^	**16.8 (**<**0.001)** ^a^	0.964 (0.326)
NE Europe: Szeged, Hungary	−5.5 (6.3)	0.4 (0.501)			−7.6 (6.1)	**12.0 (**<**0.001)** ^a^		
NE Europe: Tartu, Estonia	1.1 (4.9)	**115.7 (**<**0.001)** ^a^			0.4 (4.4)	**104.2 (**<**0.001)** ^a^		

Bold values indicate “statistical significant” results

*ANOVA* analysis of variance, *MUAC* mid-upper arm circumference

^a^ Bonferroni multiple-comparison test

**Fig. 2:**
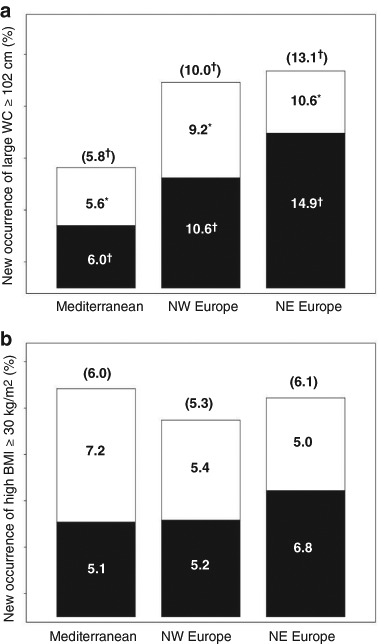
New occurrence of men with large waist circumference (**a)** or with high BMI (**b)** in cities from three regions of Europe (differences from Mediterranean region: ^*^ *P* < 0.05, ^†^ *P* < 0.01)

Figure 2a shows that in all men aged 40–79 years, over 4.3 years, the development of WC ≥ 102 cm was lowest at 5.8 % in Mediterranean cities, 10.0 % in NW European cities and highest (13.1 %) in NE European cities. Further analysis by age groups revealed that these regional differences in developing large WC were more pronounced in men aged 60–79 compared with those aged 40–59 years. There were no regional differences in changes in prevalence of BMI ≥ 30 kg/m^2^ (Fig. 2b).

Table 3 shows that compared with men living in Mediterranean cities, adjusted odds ratios for developing a WC ≥ 102 cm were 1.6-fold greater in men living in NW European cities and 2.3-fold greater in men living in NE European cities. Compared with men living in cities from non-transitional countries, the odds ratio for developing WC ≥ 102 cm was 1.6 fold greater in men living in cities from the transitional countries. There were no regional differences in odds ratios for developing a BMI ≥ 30 kg/m^2^. Odds ratios for regional differences in developing a large WC remained significant when analysed by age group, and were greater in men aged 60–79 than 40–59 (Table 4A). There were no regional differences in developing a BMI ≥ 30 kg/m^2^ in either age group (Table 4B).

**Table 3:** Logistic regression to determine odds ratios for 4.3 year development of large WC or high BMI between individual cities, between cities from the Mediterranean, NE Europe and NW Europe, and between cities from transitional and non-transitional regions

		OR (95 % CI) for development of large WC^﻿a﻿^				OR (95 % CI) for development of high BMI^a^		
	New occurrence of WC ≥ 102 cm	Between individual cities	Between Regions	Between Transitional status (non-transitional/transitional)	New occurrence of BMI ≥ 30 kg/m^2^	Between individual cities	Between Regions	Between Transitional status (non-transitional/transitional)
Mediterranean: Santiago de Compostela, Spain	4.9 %	Referent	Referent	Referent	6.6 %	Referent	Referent	Referent
Mediterranean: Florence, Italy	6.6 %	1.71 (0.82–3.77) *P* = 0.163			5.5 %	0.99 (0.48–2.08) *P* = 0.982		
NW Europe: Leuven, Belgium	11.8 %	**2.59 (1.32**–**5.50)** *P* = **0.008**	**1.58 (1.05**–**2.44)** *P* = **0.033**		5.9 %	1.20 (0.59–2.52) 0.619	0.98 (0.62–1.57) *P* = 0.929	
NW Europe: Malmö, Sweden	11.0 %	**2.35 (1.21**–**4.96) 0.016**			5.2 %	0.90 (0.44–1.88) *P* = 0.770		
NW Europe: Manchester, UK	6.7 %	1.25 (0.57–2.84)*P* = 0.581			4.7 %	0.80 (0.36–1.78) *P* = 0.580		
NE Europe: Łódź Poland	15.5 %	**3.55 (1.80**–**7.54)** *P* <**0.001**	**2.28 (1.49**–**3.54)** *P* <**0.001**	**1.60 (1.21**–**2.12)** *P* = **0.001**	7.4 %	1.34 (0.65–2.84) 0.427	1.05 (0.65–1.73) *P* = 0.833	1.17 (0.81–1.67) *P* = 0.408
NE Europe: Szeged, Hungary	13.0 %	**3.10 (1.50**–**6.85) 0.003**			6.9 %	1.21 (0.56–2.66) *P* = 0.624		
NE Europe: Tartu, Estonia	10.8 %	**2.43 (1.19**–**5.33)** *P* = **0.019**			3.7 %	0.68 (0.28–1.58) *P* = 0.367		

Bold values indicate “statistical significant” results

*BMI* body mass index, *CI* confidence interval, *OR* odds ratio, *WC* waist circumference

^a^ Adjusted for baseline lifestyle factors, socioeconomic status and physical activity level

**Table 4A:** Logistic regression to determine ORs for 4.3 year development of large WC between individual cities, between cities from the Mediterranean, NE Europe and NW Europe, and between cities from transitional and non-transitional regions in middle-aged and elderly men

	Middle aged men (40–59 years)				Elderly men (60–79 years)			
	New occurrence of WC ≥ 102 cm	OR (95 % CI) for development of large WC^a^			New occurrence of WC ≥ 102 cm	OR (95 % CI) for development of large WC^a^		
		Between individual cities	Between Regions	Between Transitional status (non-transitional/transitional)		Between individual cities	Between regions	Between Transitional status (non-transitional/transitional)
Mediterranean: Santiago de Compostela, Spain	6.0 %	Referent	Referent	Referent	4.0 %	Referent	Referent	Referent
Mediterranean: Florence, Italy	5.4 %	0.86 (0.28–2.79) *P* = 0.790			7.4 %	2.94 (1.05–9.76) *P* = 0.054		
NW Europe: Leuven, Belgium	9.0 %	1.35 (0.49–4.08) *P* = 0.573	1.69 (0.89–3.40) *P* = 0.123		13.7 %	**3.89 (1.52**–**12.04)** *P* = **0.009**	1.45 (0.84–2.58) *P* = 0.194	
NW Europe: Malmö, Sweden	13.2 %	2.50 (0.99–7.24) *P* = 0.067			9.3 %	2.16 (0.81–6.79) *P* = 0.148		
NW Europe: Manchester, UK	4.7 %	0.99 (0.29–3.39) *P* = 0.989			8.2 %	1.14 (0.38–3.88) *P* = 0.820		
NE Europe: Łódź Poland	12.9 %	2.70 (1.04–7.90) *P* = 0.050	1.86 (0.97–3.74) *P* = 0.069	1.43 (0.90–2.24) *P* = 0.123	17.0 %	**3.47 (1.30**–**11.14)** *P* = **0.021**	**2.48 (1.42**–**4.49)** *P* = **0.002**	**1.73 (1.20**–**2.48)** *P* = **0.003**
NE Europe: Szeged, Hungary	9.8 %	1.65 (0.59–5.16) 0.358			15.8 %	**4.95 (1.79**–**16.23)** *P* = **0.004**		
NE Europe: Tartu, Estonia	9.3 %	1.64 (0.54–5.37) *P* = 0.389			11.8 %	**3.08 (1.13**–**9.97)** *P* = **0.039**		

Bold values indicate “statistical significant” results

*BMI* body mass index, *CI* confidence interval, *OR* odds ratio

^a^ Adjusted for baseline lifestyle factors, socioeconomic status and physical activity level

**Table 4B:** Logistic regression to determine ORs for 4.3 year development of high BMI between individual cities, between cities from the Mediterranean, NE Europe and NW Europe, and between cities from transitional and non-transitional regions in middle-aged and elderly men

	Middle aged men (40–59 years)				Elderly men (60–79 years)			
	New occurrence of BMI ≥ 30 kg/m^2^	OR (95 % CI) for development of high BMI^a^			New occurrence of BMI ≥ 30 kg/m^2^	OR (95 % CI) for development of high BMI^a^		
		Between individual cities	Between regions	Between transitional status (non-transitional/transitional)		Between individual cities	Between regions	Between transitional status (non-transitional/transitional)
Mediterranean: Santiago de Compostela, Spain	7.7 %	Referent	Referent	Referent	5.8 %	Referent	Referent	Referent
Mediterranean: Florence, Italy	6.8 %	0.91 (0.33–2.67)*P* = 0.861			4.5 %	0.91 (0.32–2.63) *P* = 0.849		
NW Europe: Leuven, Belgium	4.7 %	0.77 (0.25–2.35)*P* = 0.635	0.93 (0.48–1.85)*P* = 0.844		6.7 %	1.43 (0.54–4.08) *P* = 0.484	0.96 (0.50–1.89) *P* = 0.904	
NW Europe: Malmö, Sweden	6.7 %	0.94 (0.34–2.71)0.906			4.1 %	0.76 (0.27–2.24) *P* = 0.613		
NW Europe: Manchester, UK	4.7 %	0.72 (0.22–2.29)*P* = 0.577			4.6 %	0.78 (0.25–2.50) *P* = 0.673		
NE Europe: Łódź Poland	3.4 %	0.52 (0.13–1.80) *P* = 0.312	0.84 (0.41–1.76)*P* = 0.646	0.89 (0.49–1.57) *P* = 0.693	9.8 %	1.84 (0.68–5.39) *P* = 0.240	1.14 (0.57–2.32) *P* = 0.718	1.33 (0.82–2.14)*P* = 0.247
NE Europe: Szeged, Hungary	7.4 %	1.35 (0.45–4.22) 0.596			6.5 %	0.98 (0.32–3.14) *P* = 0.967		
NE Europe: Tartu, Estonia	3.3 %	0.58 (0.14–2.15) *P* = 0.425			3.9 %	0.70 (0.21–2.31) *P* = 0.556		

*BMI* body mass index, *CI* confidence interval, *OR* odds ratio

^a^ Adjusted for baseline lifestyle factors, socioeconomic status and physical activity level

We have also analysed changes in the intermediate categories of adiposity (results not shown), there were only differences in those whose WC rose from the lowest to moderately elevated (94–101.9 cm) between Santiago de Compostela and Malmö, (decrease). Compared with Santiago de Compostela, the adjusted odds ratios in increase in prevalence of moderately high BMI (25–29.9 kg/m^2^) were increased in Florence, Leuven, Malmö, Łódź and Szeged.

## Discussion

We found that WC, a marker of secondary disease risk through its correlation with total body fat and with central fat accumulation in thinner subjects [24], rose substantially over 4.3 years. The prevalence of WC ≥ 102 cm (indicating high risk of metabolic disease) [1, 2], increased most in cities from NE Europe, followed by cities from NW Europe and lowest in cities from the Mediterranean. These regional differences in rising WC were most pronounced in men aged 60–79 years. While BMI rose over the study period, there were no regional differences in the rises in prevalence of high BMI, nor was there an age trend. Increased central fat accumulation among men living in cities from NE European was accompanied by significant loss of mid-upper arm muscle area.

Large WC indicates an increase in the metabolically active intra-abdominal fat which is associated with specific clusters of symptoms, secondary metabolic syndrome and chronic diseases including diabetes, hypertension and cardiovascular disease [1, 2]. These conditions are exacerbated by adverse lifestyle factors such as smoking, poor diet and physical inactivity which tend to co-exist with obesity in post-industrial communities [25]. Given the already-high baseline prevalence of large WC in men living in the transitional region, their greater risk of developing large WC over the study period is likely to herald many downstream health complications.

These data suggest that the rising adiposity with age, especially of WC (but less of BMI), appears to be more pronounced in transitional countries presumably related to the recent and continuing socio–political and economic changes. Our data were adjusted for a number of sociodemographic and lifestyle factors therefore the results may reflect the obesogenic social conditions due at least in part to sedentary lifestyles and consumption of energy-dense food [14]. We have also performed unadjusted analyses and found that differences between countries and between regions were slightly stronger than adjusted data (results not shown). However, the dissociation between WC and BMI changes may point towards an increase in individual susceptibility to weight gain and cardiometabolic risk. The lack of regional differences in BMI may be accounted for by the concurrently greater loss of mid-upper arm muscle area, which may indicate a general loss of lean body tissue, in NE European men. Age-related muscle atrophy is the central feature of sarcopenic syndrome, a condition associated with frailty and poor health [26, 27]. Sarcopenic obesity, a condition associated with excess energy consumption, sedentary lifestyle and chronic illnesses [28], is associated with even worse health outcomes [29]. We should emphasize that changes in adiposity and mid-upper arm muscle area in the present study were calculated on the whole group, rather than individual, basis.

Mackenbach et al. [12] have previously observed increased mortality in lower economic status sectors of Europe, most marked in Eastern European countries. In contrast, in Mediterranean countries which have been more socially and culturally stable for many decades [30], people have continued to consume traditional Mediterranean diets characterised by fibre-rich fruit, vegetables legumes and cereals, more fish and poultry than red meat, and moderate alcohol intake [31]. This diet is rich in monounsaturated fatty acids and in micronutrients with anti-inflammatory [32] and antioxidant properties [33]. Large prospective studies have shown that adherence to Mediterranean diet leads to a significant reduction in WC [34–36] and BMI [37], consistent with evidence which links Mediterranean diets to lower cardiovascular risk [34].

The present study also showed that developing a large WC was significantly more likely in cities from NW European than in cities from Mediterranean countries. This difference within the non-transitional zone suggests that diet and lifestyle factors are probably more influential than socioeconomic differences as risk factors for increased central fat accumulation, since NW European countries have higher gross national income per capita than Mediterranean countries [38].

In our study, regional differences in the development of large WC continued to persist after adjustments (but the adjustments did attenuate the ORs to some/small extent) for lifestyle factors including smoking and physical activity and socioeconomic status including educational attainment level and employment status. It is therefore likely that other factors contribute to these differences. There is a body of evidence indicating that in utero exposure to maternal factors such as obesity, poor diet and smoking can modify gene expression though DNA methylation and histones, which can exacerbate obesity in later life [8, 39]. It is now known that these epigenetic changes can be inherited, and their effects continue over several generations [40]. In principle, epigenetic effects may be harmful or protective. In keeping with other studies of epigenetic effects from smoking [41, 42], we have demonstrated previously that maternal smoking associates with future adult adiposity and cardiovascular risk [43]. Parental smoking history was not collected in EMAS but transitional countries had the highest prevalence of smokers and ex-smokers at baseline [9] and smoking is strongly retained as an inter-generational trait within families [43]. Thus exposure to parental tobacco smoke in early life or in utero may have driven some of the harmful epigenetic changes. It remains possible that the relatively stable Mediterranean diet, and lifestyle confers protective epigenetic effects, or that changes in social and domestic conditions during politico–economic strife induce stress and thus harmful epigenetic changes. The latter hypothesis may also apply to the increased rates of central adiposity, cardiovascular and metabolic diseases seen ubiquitously amongst migrant populations [44] which tend to live under chronic stress. Evidence from animal studies demonstrates adverse epigenetic changes after exposure to stress in pregnancy [45].

Maternal smoking, including passive smoking, and stress during pregnancy cause poor intra-uterine growth and low birthweight, which predisposes individuals to increased intra-abdominal/central fat accumulation and cardio-metabolic risk in adult life [46, 47], particularly when exposed to abundant high-energy foods [48]. Rates of low birthweight (<2500 g) were similar, ranging between 5 and 9 % among the eight countries in the present study in 2000 [49]. Birthweight data are not available for the period 1923–1963 when subjects in the present study were born, but that was a time of greater economic austerity and stress in the transitional countries, so birthweights might have been lower than in non-transitional countries.

Inevitably there are limitations to the interpretation of these data. However, the present study has particular strengths in its prospective design and detailed contemporary assessment of contrasting populations in Europe, capturing a period of considerable social change in some European countries. The study samples were representative locally, but not necessarily nationally. We enrolled mainly Caucasian men with a study response rate of 41 %, which could limit generalizability. Although the EMAS database is large, allowing assessment of many aspects of male aging, there are pieces of information which could not reliably be collected, such as birthweight and dietary assessment, and others where risk of participant-fatigue meant that self-reported information could not be complemented by measured data, such as physical activity by odometers and dietary intake. The statistical methods employed were robust, and data were adjusted where possible for potential confounders and co-variates, but residual confounding is always possible, particularly in a cross-cultural study of this kind where measures of socioeconomic status may vary. Information on women would also be of value and it would be of interest to relate the changes in adiposity to dietary and other behavioural habits.

In conclusion, while weight and BMI is generally rising among European men, WC are increasing more rapidly, particularly in countries undergoing socio–economic and political transition. This changing body shape profile predicts greater future burdens from cardio-metabolic diseases and, according to recent studies, from cancer [3, 4].
